# Fatal Disseminated Kaposi's Sarcoma due to Immune Reconstitution Inflammatory Syndrome following HAART Initiation

**DOI:** 10.1155/2013/546578

**Published:** 2013-07-07

**Authors:** Fatuma Catherine Atieno Odongo

**Affiliations:** Division of Infectious Disease, University of São Paulo, Avenida Dr. Enéas de Carvalho Aguiar, 255, 4°Andar, 05403-900 São Paulo, SP, Brazil

## Abstract

This is a case report of disseminated Kaposi's sarcoma in the context of immune reconstitution inflammatory syndrome in an HIV-infected patient on HAART regimen for 2 months. The patient rapidly progressed to death in 5 days after worsening pulmonary infiltrates and multiple organ failure.

## 1. Introduction

Kaposi's sarcoma (KS), a low-grade malignancy that is associated with human herpesvirus-8 (HHV-8), is a multifocal tumor that most commonly affects mucocutaneous sites. In its disseminated form, it can also involve lymph nodes and visceral organs, in particular the respiratory and gastrointestinal tract, but every organ can potentially be affected, including bone tissue.

Four forms of the disease have been recognized: the classic form (occurring in Mediterranean elders), the endemic form (occurring in African children), the transplant-associated form (resulting from iatrogenic immunosuppression), and the epidemic form which is associated with HIV infection.

Although antiretroviral therapy is a condition for AIDS associated KS improvement and resolution, in disseminated cases, HAART may rarely induce an immune reconstitution inflammatory syndrome (IRIS) of varying severity and even fatal cases. IRIS in the context of KS is rare and often not readily recognized, thus delaying the necessary immediate measures.

## 2. Case Report

A 29-year-old homosexual male patient was admitted at the emergency unit on February 22, 2012, complaining of shortness of breath and a dry cough for the past 3 days. He mentioned associated intermittent fever of 38°C, bloody sputum, and weight loss. 

Two months prior to the emergency room admission, he had received a positive anti-HIV ELISA test and an initial CD4+ lymphocyte count of 136 cells/mm^3^. Highly active anti-retroviral therapy (HAART) consisting of lamivudine, zidovudine and efavirenz had been initiated. 

Two weeks prior to hospital admission, he had been started on prophylactic sulfamethoxazole-trimethoprim (SMX-TMP), and on the same date, it had been noted that he presented purplish skin lesions on his anterior chest, right eyelid, right arm, and shoulder, as well as groin area, hard palate, and uvula, all suggestive of Kaposi's sarcoma (KS) in association with oral candidiasis.

Due to worsening respiratory symptoms on the second day of hospital admission, he was referred to the intensive care unit of the Department of Infectious Diseases. On admission at the ICU, he was alert and oriented, with a stable blood pressure, but with a respiratory rate of 24 breaths per minute and hypoxemia with SPO2 = 82% at room air. 

**Figure 1:**
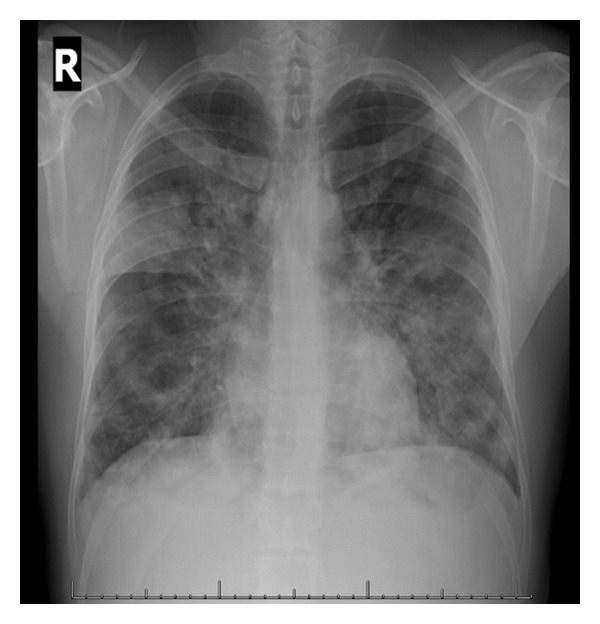
Initial chest X-ray at the ICU, showing diffuse bilateral cotton-like infiltrates.

**Figure 2:**
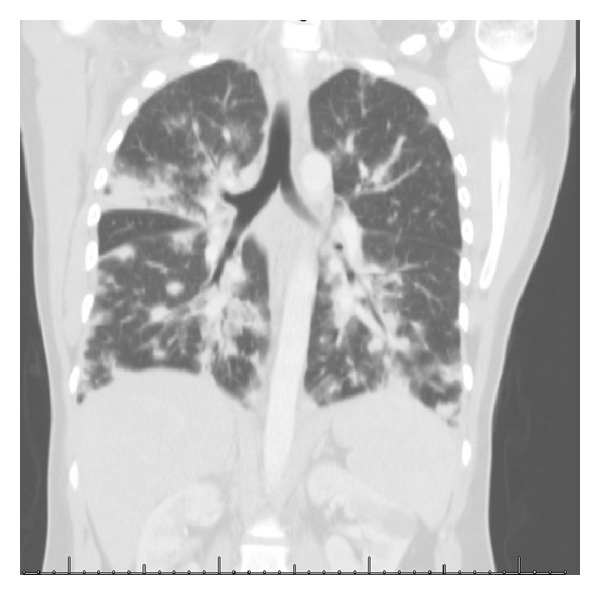
Initial chest CT scan at the ICU, revealing bilateral patchy infiltrates suggestive of lung KS or alveolar hemorrhage.

Other notable examination findings were micropolyadenopathy and hepatosplenomegaly. His initial chest X-ray showed diffuse bilateral cotton-like infiltrates (Figure 1). His chest CT scan revealed bilateral patchy infiltrates suggestive of lung KS or alveolar hemorrhage (Figure 2).

Empirical antibiotic therapy for severe community acquired pneumonia (CAP) with ceftriaxone and clarithromycin was started immediately, as well as treatment for oral candidiasis with intravenous fluconazole. Antiretroviral therapy was modified to zidovudine, lamivudine, and lopinavir with booster ritonavir. SMX-TMP was increased to its therapeutic dose for *Pneumocystis* pneumonia.

The patient was submitted to endotracheal intubation due to severe hypoxemia. Bronchoscopy revealed purplish lesions in the respiratory tract with signs of alveolar hemorrhage. A digestive endoscopy also showed purplish lesions in the duodenum and stomach. Mucosal biopsy was hindered by the risk of severe uncontrolled hemorrhage. The skin biopsy confirmed cutaneous KS.

**Figure 3:**
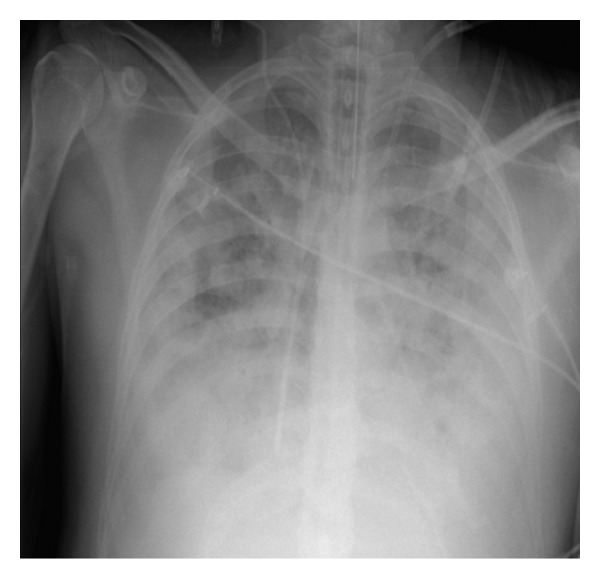
Last chest X-ray at the ICU, showing worsening diffuse bilateral nodular and patchy infiltrates.

On the fifth day of hospital, the patient presented hemodynamic instability unresponsive to vasoactive drugs (noradrenalin at 2 mcg/kg/min, adrenalin at 2 mcg/kg/min, and dobutamine at 6.6 mcg/kg/min), acute kidney failure with severe metabolic acidosis, and hyperkalemia requiring renal replacement therapy. The ordered chest X-ray at the ICU showed worsening diffuse bilateral nodular and patchy infiltrates (Figure 3). Antibacterial therapy was changed to meropenem and vancomycin to treat for possible nosocomial bacterial infection causing septic shock. Despite adequate therapeutic measures, the patient expired on the 5th day of hospital admission. 

All blood cultures were negative. The tracheal aspirate was negative for fungi, bacteria, and *Mycobacterium*, except for a positive qualitative PCR for cytomegalovirus. The patient's CD4+ lymphocyte count at the time of expiry was 391 cells/mm^3^ with a viral load of 1,353 copies/mL (Log 3.13).

## 3. Discussion

Kaposi's sarcoma (KS) is among the most common cancers in AIDS patients, especially in African countries, and occurs at all CD4 levels below 500 cells/mm^3^. The most common manifestations are purple skin lesions, prompting a thorough investigation of the pulmonary and gastrointestinal tracts for staging and therapeutics planning owing to the frequency of advanced KS at diagnosis. One case report showed that, in its disseminated form, KS can even affect the articulation [1]. 

Regarding treatment options, targeted therapy for KS should be started based on disease severity on the initial patient evaluation. Localized or minimally disseminated cutaneous KS mostly responds to highly active anti-retroviral therapy (HAART) and either surgical resection of the lesion, cryotherapy, or radiotherapy can be associated. HAART does not have direct effects on HHV-8 but causes partial or complete resolution of KS lesions through a decrease in HIV viral load and immune reconstitution of memory CD4+ lymphocyte cells. 

Disseminated disease with visceral involvement would require an association of HAART regimen with chemotherapy agents, for example, paclitaxel and liposomal doxorubicin.

Although HAART is a condition for KS improvement and resolution, in disseminated cases, HAART may rarely induce an immune reconstitution inflammatory syndrome (IRIS) of varying severity and even fatal cases [2]. In the literature, KS related IRIS (KS-IRIS) is rarely reported when compared to other more common diseases, such as tuberculosis, cryptococcal meningitis, *Mycobacterium avium-intracellulare* infection, and *Pneumocystis jirovecii* pneumonia. 

In the presented clinical case, the patient's cutaneous lesions were evident approximately 6 weeks after initiating HAART, suggesting probable unmasking of occult HHV-8 infection and subclinical KS. According to a prospective study carried out in Mozambique, risk factors for KS-IRIS include pretreatment KS (clinical or subclinical), detectable plasma HHV-8 DNA, hematocrit below 30%, and high plasma HIV viral load [3].

In general, IRIS treatment depends on the clinical severity and disease agent. Treatment of KS-IRIS is a challenge because this entity is rarely recognized and its appropriate therapeutic management is still unclear. HAART discontinuation and corticosteroid use in moderate to severe IRIS cases is a widely known measure that diminishes inflammation to varying degrees. However, KS-IRIS is not routinely treated with corticosteroids due to the risk of further compromise of cellular immunity permitting HHV-8 replication and tumor growth [4]. Ideally, it is best to prevent KS-IRIS through early recognition of disease, initial chemotherapy treatment, and later on HAART introduction. 

In one paper, it is suggested that starting or maintaining chemotherapy in association with continuing HAART may cause successful resolution on KS-IRIS. To highlight this, I exemplify Elizabeth Connick's brief report where the patient presented with facial KS and significant neck edema and lymphadenopathy while on HAART and this inflammation would resolve coincidentally with decreases in the CD4+ lymphocyte count during paclitaxel treatment. This patient KS finally cleared after prolonged HAART and chemotherapy. The authors thus concluded that clinicians should be aware of KS-IRIS and that they should realize that IRIS does not indicate failure of HAART or a need for changes in anti-retroviral regimen. Instead, chemotherapy in conjunction with HAART can effectively control the symptoms of IRIS and resolve KS [5].

## 4. Final Considerations

The patient in this case report presented with numerous skin lesions that already suggested a case of disseminated KS which was later confirmed. He was not promptly started on chemotherapy as indicated for disseminated visceral KS and this certainly may have contributed to his deteriorating clinical status and death due to KS-IRIS. Although pulmonary KS has a poor prognosis even when chemotherapy is started early, it can be concluded that chemotherapy is necessary and should be administered in pulmonary KS-IRIS due to its high mortality  rate.
